# Enhancing Anaerobic Digestion of Agricultural By-Products: Insights and Future Directions in Microaeration

**DOI:** 10.3390/bioengineering12101117

**Published:** 2025-10-18

**Authors:** Ellie B. Froelich, Neslihan Akdeniz

**Affiliations:** Biological Systems Engineering, University of Wisconsin-Madison, Madison, WI 53706, USA

**Keywords:** anaerobic digestion, microaeration, biogas production, microbial community

## Abstract

Anaerobic digestion of manures, crop residues, food waste, and sludge frequently yields biogas with elevated hydrogen sulfide concentrations, which accelerate corrosion and reduce biogas quality. Microaeration, defined as the controlled addition of oxygen at 1 to 5% of the biogas production rate, has been investigated as a low-cost desulfurization strategy. This review synthesizes studies from 2015 to 2025 spanning laboratory, pilot, and full-scale anaerobic digester systems. Continuous sludge digesters supplied with ambient air at 0.28–14 m^3^ h^−1^ routinely achieved 90 to 99% H_2_S removal, while a full-scale dairy manure system reported a 68% reduction at 20 m^3^ air d^−1^. Pure oxygen dosing at 0.2–0.25 m^3^ O_2_ (standard conditions) per m^3^ reactor volume resulted in greater than 99% removal. Reported methane yield improvements ranged from 5 to 20%, depending on substrate characteristics, operating temperature, and aeration control. Excessive oxygen, however, reduced methane yields in some cases by inhibiting methanogens or diverting carbon to CO_2_. Documented benefits of microaeration include accelerated hydrolysis of lignocellulosic substrates, mitigation of sulfide inhibition, and stimulation of sulfur-oxidizing bacteria that convert sulfide to elemental sulfur or sulfate. Optimal redox conditions were generally maintained between −300 and −150 mV, though monitoring was limited by low-resolution oxygen sensors. Recent extensions of the Anaerobic Digestion Model No. 1 (ADM1), a mathematical framework developed by the International Water Association, incorporate oxygen transfer and sulfur pathways, enhancing its ability to predict gas quality and process stability under microaeration. Economic analyses estimate microaeration costs at 0.0015–0.0045 USD m^−3^ biogas, substantially lower than chemical scrubbing. Future research should focus on refining oxygen transfer models, quantifying microbial shifts under long-term operation, assessing effects on digestate quality and nitrogen emissions, and developing adaptive control strategies that enable reliable application across diverse substrates and reactor configurations.

## 1. Introduction

Anaerobic digestion (AD) is a biological process that converts organic carbon into biogas, primarily consisting of 40–70% methane (CH_4_) and 30–60% carbon dioxide (CO_2_), along with trace amounts of water vapor and other gases [1,2]. It plays a crucial role in agricultural by-product management by treating manure, crop residues, and other organic by-products while producing renewable energy [3]. However, a persistent challenge in AD is the presence of hydrogen sulfide (H_2_S) in biogas, especially when digesting sulfur-rich substrates such as manure and agro-industrial wastes. H_2_S concentrations can range from a few hundred to several thousand parts per million (ppm), causing severe corrosion in engines and infrastructure [4].

Traditional H_2_S removal methods (iron salts dosing, scrubbers, activated carbon, etc.) can be costly or complex, prompting interest in in situ biological desulfurization techniques [5]. One such strategy is microaeration, which involves injecting a small amount of air or pure oxygen into an AD system to foster the activity of sulfur-oxidizing bacteria (SOB) present in the digester. These facultative or microaerobic microbes oxidize dissolved sulfide to elemental sulfur or sulfate, thereby reducing H_2_S formation [6,7].

This desulfurization method is appealing for farm-based digesters due to its simplicity and low cost, as only a small amount of airflow (typically 1–5% of the biogas volume) is required [5,8]. In addition to H_2_S removal, carefully controlled microaeration can enhance overall AD performance. Recent studies, particularly over the last decade, have indicated that microaeration can enhance the hydrolysis of complex substrates, increase methane yields, and stabilize the digestion process [6,9,10].

These benefits may seem counterintuitive, as oxygen typically inhibits strict anaerobes, such as methanogenic archaea. The key is that at very low doses, oxygen is quickly consumed by facultative bacteria, preventing a complete shift to aerobic conditions while still positively altering the microbial community and biochemical pathways [7].

Over the past decade, research on microaeration in AD has expanded across laboratory reactors, pilot projects, and full-scale digesters. Agricultural applications are of particular interest: for example, dairy or pig manure digesters often produce biogas with high H_2_S levels (3000–5000 ppm) and could benefit substantially from on-site H_2_S mitigation [11,12].

Lignocellulosic agricultural residues that are resistant to biodegradation may also show improved breakdown under microaerobic conditions [13]. Numerous laboratory studies, pilot projects, and full-scale trials have evaluated various microaeration techniques, dosages, and control strategies. Reported outcomes include significant H_2_S reductions (often exceeding 90% removal), moderate increases in CH_4_ production (typically 5–20%), faster substrate hydrolysis, and microbial community shifts that favor process stability [6,9,12,14].

On the other hand, excessive or mismanaged aeration can negatively impact CH_4_ yields by diverting substrate to CO_2_ or inhibiting obligate anaerobes, and poses safety risks as excess oxygen in biogas can create explosive mixtures [11,15,16]. Thus, it is critical to evaluate both the advantages and risks of microaeration under different operating conditions.

This literature review provides a comprehensive overview of scientific research on microaeration in AD systems over the last decade (approximately 2015 to 2025). We focus primarily on agricultural waste digestion, while also drawing lessons from studies on sewage sludge, food waste, and other substrates. Key areas addressed include: (1) the range of microaeration techniques and implementation methods, (2) impacts on CH_4_ production, biogas composition (H_2_S reduction and other metrics), and overall digester performance, (3) the underlying mechanisms and microbial community responses to microaeration, and (4) practical considerations, case studies, and comparisons of benefits and drawbacks of microaeration in different AD systems. By synthesizing findings from recent studies, this review aims to guide both researchers and practitioners in understanding how and when microaeration can improve AD and what design or operational factors must be considered for successful application.

While previous reviews [7,8] summarized the general mechanisms, microbial interactions, and process control strategies of microaeration across diverse waste streams, the present review differs by focusing specifically on agricultural by-products, including manures and lignocellulosic residues, whose intrinsic characteristics strongly influence the effects of microaeration. High lignin content limits hydrolysis but can be partially modified under trace oxygen exposure, improving enzymatic access to cellulose and hemicellulose [17]. Elevated C/N ratios and variable sulfur contents in agricultural substrates alter redox balance, oxygen demand, and microbial community dynamics [18]. Moisture content, particle size, and trace mineral composition further shape oxygen transfer efficiency and the formation of localized micro-zones [19]. By examining these substrate-specific factors, this review highlights how microaeration principles must be adapted for agricultural systems to achieve consistent methane yields and effective hydrogen sulfide mitigation.

While several emerging approaches, such as biochar-supported desulfurization and membrane-aerated biofilm systems, have been explored for hydrogen sulfide control, their costs, operational complexity, and scalability remain limiting at full scale [20,21]. In contrast, microaeration offers a simple, low-cost in situ alternative that can be readily incorporated into existing anaerobic digesters, making it a distinctive and practical pathway for improving biogas quality.

**Figure 1:**
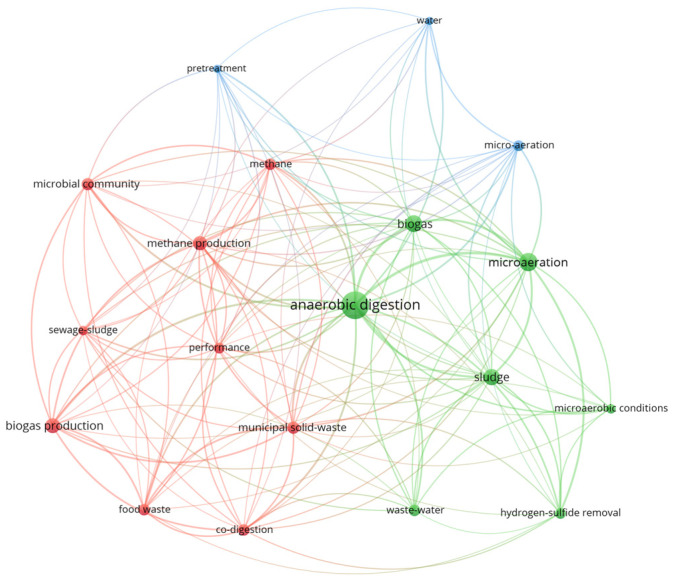
Keyword co-occurrence map focusing on anaerobic digester studies published in the last ten years (2016–2025). Different colors represent distinct clusters of closely related keywords.

The co-occurrence map below (Figure 1) illustrates the research landscape surrounding anaerobic digestion, highlighting three interconnected areas. The map was prepared using VOSviewer (version 1.6.20), focusing on the studies published in the last 10 years (2016–2025). At the center, anaerobic digestion links studies focused on process control, microbial activity, and substrate utilization. The green cluster emphasizes process enhancement through microaeration, biogas quality improvement, and hydrogen sulfide removal, reflecting growing interest in controlled oxygen introduction to stabilize digestion and enhance biogas purity. The red cluster centers on feedstock management and methane generation, connecting microbial community dynamics with co-digestion of food waste, sewage sludge, and municipal solid waste. The blue cluster represents upstream system integration and pretreatment, indicating interest in combining microaeration with preprocessing strategies to improve hydrolysis and overall conversion efficiency. Together, these clusters demonstrate that current research is increasingly integrating biological, chemical, and operational approaches to improve the stability and efficiency of anaerobic systems, while enhancing biogas yield and quality.

## 2. Overview of Anaerobic Digestion Systems

### 2.1. Operational Scales of Anaerobic Digestion

AD systems are implemented at various operational scales, each serving distinct purposes in research, development, and practical application. Laboratory-scale digesters are typically small, controlled reactors designed to investigate fundamental processes, microbial interactions, and substrate behavior under tightly regulated conditions. These systems offer the advantage of rapid experimentation and cost efficiency, but may not fully replicate the complexities found in larger operations [22]. Pilot-scale digesters bridge the gap between lab studies and real-world applications, offering a mid-sized platform to evaluate process performance, optimize operational parameters, and identify potential scale-up challenges. They often simulate the conditions of full-scale plants more accurately while still allowing flexibility for adjustments [22,23]. Full-scale digesters represent commercial or industrial installations designed for continuous waste treatment and biogas production. These large systems operate under practical constraints such as substrate variability, hydraulic retention times (HRT), and economic considerations, providing essential data on long-term stability, efficiency, and environmental impact in real-world conditions [7,24].

### 2.2. Batch Systems

Batch AD systems operate by loading all the feedstock into the reactor at the beginning of the process and sealing the system to proceed without further input or output until digestion is complete. This configuration is common in laboratory experiments due to its simplicity, low cost, and ease of control. Batch systems are often used to assess substrate-specific CH_4_ potential, microbial succession, or test the effects of additives such as oxygen in microaeration trials [25,26].

Despite their usefulness in controlled settings, batch systems have inherent limitations for continuous biogas production. Substrate depletion over time leads to declining microbial activity and gas yields, which limit their efficiency for large-scale or long-term operations [8,27]. To address this, multiple batch reactors may be operated in parallel or sequentially to approximate continuous conditions. However, the absence of regular feeding and mixing makes batch systems less suitable for evaluating long-term process dynamics or stable operational performance under real-world conditions [28].

### 2.3. Semi-Continuous Systems

Semi-continuous AD systems are widely used in both pilot-scale and full-scale operations due to their balance between operational simplicity and process stability. In these systems, fresh substrate is added and digestate is removed at regular intervals (daily, hourly, or based on set HRT) [28]. This approach avoids the instability seen in batch systems while remaining less complex than fully continuous setups. Semi-continuous feeding helps to stabilize microbial activity and gas production by preventing sharp fluctuations in loading and pH [29,30].

For microaeration, semi-continuous systems provide an ideal platform for controlled oxygen delivery. Oxygen or air can be added intermittently, in sync with feeding events or according to redox thresholds. This dosing flexibility supports the formation of microaerobic zones without the risk of prolonged exposure [31,32]. However, achieving consistent results still depends on careful tuning of aeration timing, location, and rate (particularly in minimally mixed reactors). These systems are valuable for research and on-farm applications where resource constraints or design limitations may prevent fully automated control [7,8,33].

### 2.4. Continuous Systems

Continuous AD systems represent the most technically advanced configuration, featuring uninterrupted feeding and digestate removal. These systems are often equipped with automated monitoring and control tools, allowing for consistent environmental conditions and high process efficiency [22,34]. Common in municipal wastewater treatment and large industrial biogas plants, continuous systems can operate at steady-state for extended periods, making them ideal for assessing the long-term effects of process modifications like microaeration [32,35].

The integration of microaeration into continuous systems requires sophisticated aeration control and real-time monitoring. Because the flow and mixing are constant, the introduced oxygen is quickly distributed throughout the reactor, increasing the risk of aerobic zones if dosing is not carefully calibrated [36,37]. Advanced tools (feedback-controlled sparging, oxidation-reduction potential (ORP)-regulated valves, dynamic modeling, etc.) can help maintain microaerobic conditions without disrupting CH_4_ production. These systems offer the best opportunity for scaling up microaeration, but they also demand the highest level of precision in both design and operation [31,36].

### 2.5. Operating Temperatures in Anaerobic Digestion

AD systems typically operate under two principal temperature regimes: mesophilic (30 °C to 40 °C) and thermophilic (50 to 60 °C). Mesophilic digestion is more widely used due to its greater process stability, lower energy demand, and the resilience of microbial consortia under moderate conditions [38,39]. This regime supports a diverse and robust microbial community capable of handling a wide range of feedstocks and load fluctuations, making it well-suited for municipal and agricultural applications. Additionally, mesophilic systems are less sensitive to inhibitory compounds and are easier to manage, especially in rural or decentralized settings [39].

Thermophilic digestion offers advantages in terms of faster reaction rates, higher CH_4_ yields, and improved pathogen destruction. These benefits make it attractive for applications where higher processing capacity or enhanced sanitation are desired, such as food and industrial waste treatment [39,40,41]. However, thermophilic systems are more energy-intensive, require more precise temperature control, and are often more susceptible to process instability due to a narrower range of microbial tolerance [40,41]. The choice between mesophilic and thermophilic operations depends on substrate characteristics, energy availability, and operational goals [7,8,42].

## 3. Defining Microaeration and the Microaerobic Environment

### 3.1. Definition and Historical Context

Microaeration refers to the controlled introduction of small amounts of oxygen into AD systems, typically at concentrations insufficient to support full aerobic metabolism. Historically, AD has been defined by the absence of oxygen, but research over the past two decades has revealed that carefully managed aeration can improve certain aspects of digester performance [21,43]. Originally explored as a means to reduce H_2_S concentrations in biogas, microaeration has since been investigated for broader benefits, including enhanced hydrolysis, improved process stability, and greater CH_4_ yields under specific conditions [6,9,10].

### 3.2. Characteristics of the Microaerobic Zone

**Figure 2:**
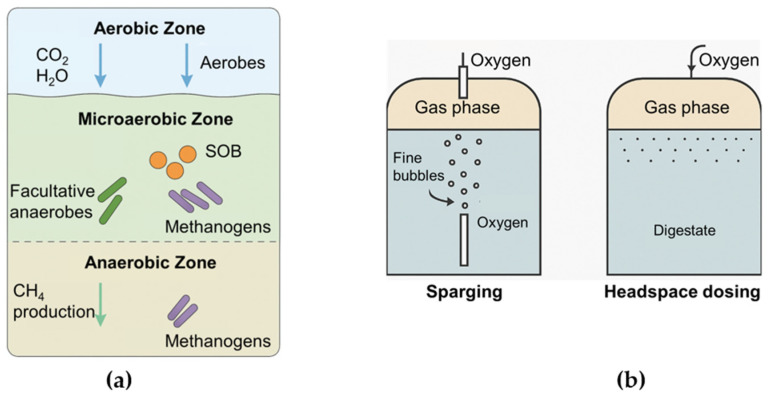
Anaerobic digestion systems: (**a**) Microbial stratification across aerobic, microaerobic, and anaerobic zones, (**b**) Oxygen delivery through sparing and headspace dosing.

In fully aerobic systems, oxygen is abundant, leading to complete oxidation of organic matter, with little to no CH_4_ production (Figure 2a). In contrast, strict anaerobic systems lack oxygen entirely, requiring syntrophic and methanogenic pathways for energy recovery [44]. The microaerobic environment is a narrowly defined ecological niche where oxygen is present in trace amounts, insufficient for aerobic dominance, but adequate to support microaerobic and facultative organisms [45]. Within an AD system, these zones are typically established near gas–liquid interfaces or via controlled oxygen sparging. The ORP in these zones is higher than in strictly anaerobic regions but remains below levels where obligate aerobes outcompete anaerobes. These conditions create a layered microbial ecosystem where oxygen-sensitive methanogens can coexist with SOB and facultative fermenters, enabling simultaneous oxidative and reductive biochemical processes [7,31,46].

Physically, microaerobic zones are dynamic and localized, and their formation depends on several factors, including reactor design, mixing intensity, aeration method, and the physical-chemical properties of the substrate. These zones may fluctuate over time, especially in high-solids systems or under variable loading conditions [8,43,47]. Maintaining consistent microaerobic conditions requires careful process monitoring, often using ORP or dissolved oxygen (DO) probes, though these metrics can be challenging to interpret in anaerobic matrices [36,37]. Understanding the spatial and temporal dynamics of these zones is essential for optimizing microbial activity and avoiding unintended consequences like excessive oxygen exposure or process imbalance.

Accurate measurement of oxygen in microaerobic environments remains technically challenging due to sensor drift, limited resolution, and delayed response in sludge-rich matrices. Conventional DO probes often drift over time and require frequent cleaning or recalibration, which can compromise data quality during long-term monitoring [48]. Emerging tools such as optical DO sensors provide higher precision and are less susceptible to biofilm accumulation, while microelectrodes allow localized profiling of dissolved oxygen gradients within granules or flocs [49]. In addition, on-line redox monitoring using oxidation–reduction potential (ORP) probes offers a reliable indirect indicator of system status, particularly when combined with automated control of aeration intensity [36]. These developments enhance process control and enable a more consistent definition of microaerobic zones in operational digesters.

## 4. Oxygen Dynamics and System Design Considerations

### 4.1. Oxygen Tolerance in Anaerobic Consortia

Anaerobic microbial consortia are traditionally considered oxygen-sensitive, particularly methanogens, which are strict anaerobes. However, many members of the anaerobic digestion community, including hydrolytic and fermentative bacteria, are facultative or aerotolerant, capable of surviving brief or low-level oxygen exposures [7,44]. The key to successful microaeration lies in exploiting this differential tolerance: low concentrations of oxygen can suppress sulfide-producing organisms or stimulate hydrolytic activity without permanently damaging methanogenic populations. Studies show that methanogens can recover from short-term oxygen stress, especially when the system maintains an overall reducing environment. The resilience of anaerobic consortia under controlled microaerobic conditions enables a strategic balance between oxidative benefits and the preservation of core anaerobic functions [7,46].

### 4.2. Oxygen Transfer Methods

Delivering small amounts of oxygen into an AD system requires carefully selected transfer methods to ensure distribution without overexposure. Two primary methods are direct sparging and headspace dosing (Figure 2b). In sparging, fine bubbles are introduced into the liquid phase, providing targeted aeration that encourages localized microaerobic zones. Sparging offers high transfer efficiency and allows for more targeted dosing, particularly in reactors with good mixing. This method is well-suited for continuous stir tank reactors or other mechanically mixed systems, where oxygen can be dispersed evenly without accumulating. However, sparging also carries risks: if not precisely controlled, it can lead to over-aeration, local inhibition of methanogens, or increased foaming. Fine control of flow rates and bubble size is therefore essential to maintain the desired oxygenation level [31,32,50].

In contrast, headspace dosing introduces oxygen into the gas phase above the digestate, relying on diffusion across the gas–liquid interface to establish microaerobic conditions. This method is especially advantageous in systems where direct sparging is impractical, such as covered lagoon digesters or plug-flow reactors with minimal mixing [33,51]. While headspace dosing generally results in slower and less uniform oxygen distribution, it tends to have a reduced risk of localized oxygen toxicity. Its passive nature also makes it more energy efficient, though achieving consistent oxygen levels can be more difficult [7,8]. For both methods, reactor design, substrate characteristics, and mixing regime significantly influence oxygen transfer dynamics and the formation of functional microaerobic zones [52].

### 4.3. Process Controls and Monitoring

Effective microaeration depends on precise control and real-time monitoring to ensure that oxygen levels remain within a narrow window that benefits the system without disrupting the digestion process. ORP and DO are two key parameters that are commonly used to characterize and control redox conditions. ORP provides an integrated measure of the system’s electron activity and is often preferred in anaerobic environments [5,21]. Typical microaerobic conditions correspond to ORP values ranging from −300 mV to −150 mV, a range that supports sulfide oxidation without compromising methanogenesis. Because ORP reflects the balance of multiple redox-active compounds, it can be used as a proxy for system health [42,53].

In contrast, DO sensors provide direct measurements of DO concentrations, but they present practical challenges in anaerobic digesters. At the trace concentrations relevant for microaeration, standard DO sensors often lack the resolution or sensitivity needed for reliable readings [42,49]. A major limitation is sensor fouling (the accumulation of sludge, biofilms, or particulate matter on the sensor surface), which can obstruct oxygen transfer to the sensor or interfere with signals. This buildup reduces accuracy and may cause delays in detecting real-time changes [48,54]. Despite these limitations, DO monitoring can be valuable when paired with ORP, particularly in well-mixed systems or at pilot scale. Advanced control systems can integrate both ORP and DO feedback to dynamically regulate aeration, using automated valves or variable flow sparging systems. Emerging technologies, including machine learning-based control, optical DO sensors, and hybrid sensor fusion, offer promising avenues to improve monitoring precision and operational resilience in microaerobic digestion systems [55,56].

## 5. Microbial Community Responses

Recent advances in high-throughput sequencing and meta-omics approaches have provided deeper insight into how microaeration restructures microbial communities and functional pathways in anaerobic digesters [6,9]. Metagenomic and metatranscriptomic analyses have identified the enrichment of sulfur-oxidizing genera such as *Thiobacillus*, *Sulfurimonas*, and *Sulfuricurvum* and the activation of *sox*, *dsr*, and *apr* gene clusters associated with sulfur oxidation and reduction under fluctuating redox conditions [9,57,58]. Concurrently, transcriptomic studies have shown upregulation of oxidative stress defense enzymes and methanogenesis-related genes (*mcrA*, *hdr*, *ftr*), indicating the resilience of hydrogenotrophic methanogens following transient oxygen exposure [15,59]. These findings highlight how controlled oxygen dosing can be tuned to sustain process stability while promoting sulfur conversion.

### 5.1. Impacts on Hydrolytic and Fermentative Bacteria

Microaeration can positively stimulate hydrolytic and fermentative bacteria, which are essential for the breakdown of complex organic compounds in AD [9]. These facultative and aerotolerant organisms can exploit trace oxygen to enhance enzymatic activity, accelerating the depolymerization of macromolecules such as carbohydrates, proteins, and lipids. This mild oxidative stimulation can improve hydrolysis efficiency, particularly in systems processing lignocellulosic substrates or manure rich in particulate organic matter. However, if oxygen levels rise too high, obligate anaerobes may become inhibited, potentially stalling downstream digestion stages [60,61].

### 5.2. Sulfur-Oxidizing Bacteria and Their Roles

One of the most beneficial microbial responses to microaeration is the proliferation of SOB, such as *Thiobacillus*. These organisms utilize trace amounts of oxygen to oxidize H_2_S into elemental sulfur (S) or sulfate (SO_4_), reducing both the concentration of corrosive gases in the biogas and the overall sulfur load in the digester. This biological oxidation pathway offers a cost-effective alternative to chemical scrubbing and is particularly effective in manure-based digesters, where the sulfur content is elevated [57,58,62]. The activity of SOB is highly dependent on the spatial and temporal availability of O_2_, often thriving in microaerobic niches, near gas–liquid interfaces, or in intermittently aerated systems [21,31]. In parallel, microaeration also influences the metabolic behavior of facultative fermenters, shifting fermentation pathways toward more oxidized intermediates like acetate and lactate while reducing hydrogen output [7]. The changes can improve downstream methanogenesis by lowering sulfide inhibition and enhancing substrate availability. However, excessive or poorly distributed oxygen may suppress strict anaerobes involved in secondary fermentation, leading to volatile fatty acid (VFA) accumulation and system instability [8,61].

Beyond direct sulfide oxidation, the enrichment of SOB under microaerobic conditions can indirectly stabilize the digestion process by modulating redox balance and intermediate metabolism. As sulfide concentrations decline, toxicity toward key fermentative and syntrophic bacteria decreases, enabling more efficient conversion of organic acids to acetate and hydrogen [6]. Several studies have reported that elevated SOB activity coincides with lower VFA accumulation and a more balanced acetogenic–methanogenic interaction, suggesting that sulfide removal alleviates inhibition on hydrogen-consuming partners and maintains syntrophic coupling [62]. The resulting redox environment favors steady methane formation and reduces the likelihood of process upsets typically associated with excess VFA buildup. This mechanistic linkage highlights the ecological role of SOB not only in biogas purification but also in preserving overall digester stability.

### 5.3. Methanogens Under Microaerobic Stress

Methanogenic archaea, particularly obligate anaerobes such as Methanosaeta and Methanobacteria, are highly sensitive to oxygen. However, studies show that many can survive short-term or low-level oxygen exposure, often by retreating into biofilms or sludge flocs that offer physical protection [59,63]. Acetoclastic methanogens tend to be more oxygen-sensitive than hydrogenotrophic types, potentially shifting the community balance under microaerobic stress [64,65]. Despite this, well-managed microaeration rarely causes complete methanogenic inhibition. Instead, a decrease in methanogenic activity may be observed during oxidation, followed by recovery, once redox conditions normalize. Long-term resilience often depends on community structure and digester configuration [15,20].

### 5.4. Microbial Community Shifts and Syntrophic Partnerships

Microaeration can trigger substantial shifts in the microbial community, often increasing overall diversity and enriching specific functional guilds. For instance, facultative fermenters may gain dominance during low-oxygen phases, temporarily outcompeting strict anaerobes [9,66]. Syntrophic partnerships, such as those between fermentative bacteria and hydrogenotrophic methanogens, may become destabilized under oxidative stress but can be reestablished as anaerobic conditions return [65]. Importantly, repeated or controlled microaeration can lead to a stable coexistence of aerobic, facultative, and anaerobic populations, facilitating parallel pathways for sulfur oxidation, VFA degradation, and CH_4_ production. These shifts underscore the need for tailored aeration strategies to maintain system resilience and metabolic activity [6,9,10].

## 6. Effects on Biogas Yield and Digester Performance

### 6.1. Effects on Methane Yield

The impact of microaeration on CH_4_ yields is closely tied to the substrate characteristics, digester type, and the rate and duration of the aeration delivery, as seen in Table 1. Food waste, a common high-strength substrate in anaerobic digestion, has shown variable but often positive responses to microaeration. In batch reactors, the response of food waste has been inconsistent, with one study reporting up to a 45.6% increase in CH_4_ production following the application of 5 L air/h for 24 h [67] while another study using a higher specific aeration rate of 400 L air/kg VS/h over the same duration observed a more modest increase of up to 4.9% [68]. Both studies were conducted under mesophilic conditions. In a semi-continuous system, a low daily aeration rate of 5 mL air/day led to a CH_4_ yield increase of up to 13.2% [69], demonstrating that even minimal oxygen inputs can enhance CH_4_ production under semi-continuous feeding conditions. No studies were identified that evaluated the effect of microaeration on food waste digestion under continuous flow conditions. Overall, these findings indicate that microaeration can enhance CH_4_ production from food waste across different reactor types and aeration strategies, but the extent of the improvement appears highly dependent on aeration rate and system configuration. Similar trends have also been observed in lignocellulosic substances.

Lignocellulosic substrates like corn, wheat, and rice straws generally benefit from controlled microaeration. In batch systems, reported effects on CH_4_ production have been highly variable, reflecting differences in oxygen dosing strategies, digestion temperatures, and experimental conditions. For example, one thermophilic study using aeration rates between 2.5 and 20 mL air/day observed CH_4_ yield improvements ranging from 1.6% to 16.1% [20]. In contrast, other batch studies conducted under mesophilic conditions reported mixed results. One study applying a high specific rate of 0.5 L air/kg/min over 2 to 8 days observed outcomes ranging from a 24.9% decrease to a 16.0% increase in CH_4_ production [67]. Similarly, another mesophilic batch experiment using rates between 0.05 and 1.6 mL air/g VS/day reported changes in the methane yield from an 11.2% decrease to a 7.8% increase [70]. In a semi-continuous system, microaeration at 0.2 mL air/g VS/day resulted in up to a 10% increase in CH_4_ production [71]. No studies were identified that evaluated microaeration effects on straw digestion in continuous flow systems. These findings underscore the importance of precise oxygen control in lignocellulosic systems, where the balance between enhanced hydrolysis and microbial inhibition is particularly delicate.

In contrast, manure-based substrates, such as cow, chicken, and swine manures, respond to microaeration in ways that reflect their distinct chemical profiles, particularly their higher ammonia (NH_3_) and sulfur content. All available studies for manure digestion were conducted under mesophilic conditions. In batch systems, microaeration generally resulted in positive effects on CH_4_ production. One study applying aeration doses ranging from 7 to 50 mL air/g VS reported CH_4_ yield increases ranging from 4.7% to 76.3% [72]. Another batch study reported a 32% increase in CH_4_ following an oxygen dose of 7.3 mL O_2_/g VS [10], while an additional batch experiment using aeration rates ranging from 12.5 to 62.5 mL air/min reported 5.7% to 13.1% increases in CH_4_ generation [14]. In semi-continuous operation, the CH_4_ response was more variable, with one study applying 33.3 mL air/L/min for 1 to 120 min, reporting changes ranging from a 20.5% decrease to a 7.9% increase [73]. In continuous systems, both studies reported positive responses. One applied 1 mL O_2_/min for 24 h every 2 days and observed up to a 22.6% increase in CH_4_ production [74], while another study reported a 21.5% increase using an aeration rate of 375 mL air/day [75]. These improvements suggest that moderate microaeration can mitigate sulfide toxicity and enhance microbial activity in manure-rich systems. However, responses vary depending on dosing strategy and manure composition.

In comparison, sewage sludge and wastewater substrates exhibit a wider range of outcomes under microaerobic conditions. All available studies for wastewater and sludge digestion were conducted under mesophilic conditions. Batch studies have reported variable CH_4_ production responses depending on aeration rates and durations. For example, one study applying 0.65 L air/L/min for 5 to 7 days observed CH_4_ increases between 31.6% and 78.9% [76], while another study using 0.5 L air/kg/min for 4 to 12 days showed results ranging from a 33% decrease to a 24.9% increase in CH_4_ production [77]. Additional batch experiments reported CH_4_ yield changes from a 12.7% decrease to a 7.2% increase at 5 to 15 mL O_2_/g VS [78], increases of 3.5% to 17.8% at 1 to 6 air volumes/g TS/min [79] and modest increases of 0.4% to 5.2% at 7.5 to 120 mL air/g COD [80]. Semi-continuous studies reported largely positive effects, with aeration doses of 5 to 150 mg O_2_/L yielding CH_4_ increases from 39.6% to 50.7% [81] and daily oxygen inputs of 29 to 82 mL O_2_/L, leading to 13.1% to 21.2% improvements [82]. No continuous system studies were identified for microaeration of wastewater or sludge. These findings highlight the sensitivity of sludge-based systems to aeration intensity, where benefits may be offset by inhibition if dosing exceeds the microbial community’s tolerance. This underscores the importance of substrate-specific aeration strategies.

Across the reviewed studies, the effectiveness of microaeration in enhancing CH_4_ production is closely tied to both reactor configuration and digestion temperature. Batch systems dominate in the literature and often report positive outcomes, though responses vary widely depending on dosing strategies and substrate complexity [67,70,72,77,78]. Semi-continuous and continuous systems appear to benefit from more stable or sustained oxygen delivery, with generally more moderate but consistent improvements. Nearly all studies were conducted under mesophilic conditions, with only one study on thermophilic digestion identified in the dataset [20]. This highlights a gap in our understanding of microaeration performance under thermophilic regimes, where microbial communities and oxygen tolerance may differ significantly. While substrate characteristics remain important, these findings emphasize that optimizing microaeration requires careful consideration of reactor type and operating temperature to balance process stability with CH_4_ yield gains.

The observed variability in methane yield across reactor configurations largely stems from differences in oxygen transfer efficiency and microbial adaptation [8]. In batch systems, limited mixing often results in transient aerobic microzones, leading to inconsistent sulfide oxidation and partial inhibition of methanogens [47]. By contrast, semi-continuous and continuous reactors maintain more stable redox gradients, leading to steadier methane yields despite smaller relative gains [74,83]. Heterogeneity in substrate composition also contributes to the wide response range [17,18]. Lignocellulosic materials exhibit slower oxygen diffusion and higher lignin resistance [19], while manure-based substrates display stronger redox buffering due to ammonia and sulfur species that neutralize oxygen stress [12,72].

Process control is crucial to prevent a reduction in CH_4_ production during microaeration. Under trace oxygen exposure, a portion of the readily biodegradable organic carbon is aerobically oxidized by facultative microorganisms, producing small additional amounts of CO_2_ that can increase its concentration in biogas by approximately 2–5% relative to strictly anaerobic conditions [8]. This effect is generally offset by improved CH_4_ formation because reduced sulfide inhibition enhances methanogenic activity, resulting in little net change in the CH_4_/CO_2_ ratio. Most of the introduced oxygen is rapidly consumed near gas–liquid interfaces or within biofilms, and only a minor fraction typically remains unreacted when aeration is well controlled [21]. If aeration exceeds microbial demand or gas mixing is insufficient, oxygen can accumulate in the headspace and increase the explosion risk when concentrations approach 4–12% v/v, the lower and upper flammability limits for CH_4_-O_2_ mixtures [84]. To prevent this, operational safeguards such as oxidation–reduction potential (ORP) feedback control (maintaining -300 to -150 mV), continuous gas monitoring for O_2_, CH_4_, and CO_2_, and automated aeration shutdown when O_2_ surpasses 2–3% are widely recommended [21,42]. These measures, combined with the natural oxygen scavenging capacity of the microbial consortium, maintain both biogas safety and composition stability during sustained microaeration.

**Table 1:** Summary of effects of microaeration on methane production during anaerobic digestion under various conditions.

Reactor Type	Materials	Digestion Temperature	Working Volume	Aeration Rate	Effect on CH_4_	Reference
Batch	Olive mill wastewater	38 °C	2 L	0.65 L air/L/min for 5–7 days	31.58–78.95% increase	[76]
Batch	Corn straw	55 °C	200 mL	2.5–20 mL air/day	1.6–16.1% increase	[20]
Batch	Municipal solid waste	35 ± 1 °C	---	0.5 L air/kg/min for 4–12 days	33% decrease-24.9% increase	[77]
Batch	Synthetic food waste	35 ± 1 °C	1 L	5 L air/h for 24 h	8.75% decrease-45.62% increase	[85]
Batch	wastewater and straw	55 °C	200 mL	5–15 mL O_2_/gVS	12.7% decrease-7.2% increase	[78]
Batch	Rice straw	25–45 °C	10 L	0.5 L/min/kg for 2–8 days	24.89% decrease-16.04% increase	[67]
Batch	Orange peel	35 ± 0.5 °C	---	400 L air/kg/h for 24 h	1.01–4.91% increase	[68]
Batch	Sludge	35 ± 1 °C	2.5 mL	1–6 air volume/gTS/min	3.5–17.8% increase	[79]
Batch	Chicken manure	37 ± 1 °C	120 mL	7–50 mL air/gVS	4.7–76.3% increase	[72]
Batch	Buffalo manure	35 ± 2 °C	600 mL	7.3 mL O_2_/gVS	32% increase	[10]
Batch	Corn straw	37 °C	200 mL	0.05–1.6 mL air/gVS/day	11.2% decrease-7.8% increase	[70]
Batch	Cow manure	35 ± 1 °C	0.8 L	12.5–62.5 mL air/L/min	5.7–13.1% increase	[14]
Batch	Sludge and food waste	35 ± 1 °C	50 mL	7.5–120 mL air/gCOD	0.4–5.2% increase	[80]
Semi-continuous	Blackwater	22 °C	1.5 L	5–150 mg O_2_/L	39.6–50.7% increase	[81]
Semi-continuous	Swine wastewater	35 ± 1 °C	4 L	33.3 mL/L/min for 1–120 min	20.5% decrease-7.9% increase	[73]
Semi-continuous	Corn straw	37 ± 1 °C	2 L	0.2 mL air/gVS	6–10% increase	[71]
Semi-continuous	Food waste	35 ± 1 °C	0.7 L	5 mL air/day	13.2% increase	[69]
Semi-continuous	Sludge and food waste	35 ± 1 °C	8 L	29–82 mL O_2_/L/day	13.1–21.1% increase	[82]
Continuous	Grass and cattle manure	35 ± 1 °C	2 L	1 mL O_2_/min for 24 h	11.76–22.55% increase	[74]
Continuous	Straw and poultry litter	37 °C	500 mL	375 mL air/day	21.5% increase	[75]

### 6.2. Effect on Hydrogen Sulfide Production

The impacts of microaeration on H_2_S concentrations during AD are most well-documented for wastewater and sludge-based substrates, which show consistently high removal efficiencies across a range of oxygen delivery rates. As shown in Table 2, many studies used ambient air dosing for their microaeration treatments. In batch systems, substantial reductions in H_2_S were observed, with one study reporting 73% to 99% removal using 1 L air/day [86] and another showing 35% to 80% removal at an aeration rate of 0.2 mg air/L [87]. While no semi-continuous studies using ambient air were identified for wastewater or sludge substrates, many continuous flow systems have been evaluated. Reported removal efficiencies ranged from moderate to near-complete, depending on the airflow rate and operational duration. For example, three studies applying 0.28 to 14 m^3^ air/h achieved up to 99.1% removal [46,88,89]. Similarly, two studies applying 2 to 400 mL air/min reported removals ranging from 60.6 to 99% removal [90,91]. Additional continuous studies using 4.4 to 6.2 NL air/m^3^/day and 8.2 to 32.7 m^3^ air/day reported 90% and greater than 99% H_2_S removal, respectively [92,93]. All but one study was conducted under mesophilic conditions. A single thermophilic study applying 0.09 to 0.77 kNm^3^ air/day observed 75–100% removal [94].

Fewer studies using pure O_2_ dosing have been conducted on wastewater or sludge-based substrates. No batch trials were identified and only one semi-continuous and one continuous system have been reported, both under mesophilic conditions. Despite the limited data, these systems have demonstrated strong H_2_S removal performance under controlled oxygen addition. In a semi-continuous study, an oxygen dose of 0.14 mL O_2_/s led to up to a 99% reduction in H_2_S [36]. A continuous flow system using 0.2 to 0.25 Nm^3^ O_2_/m^3^ reactor volume achieved greater than 99% H_2_S removal [95]. These findings suggest that sludge-based systems tolerate a wide range of aeration strategies, particularly with ambient air, while still achieving high H_2_S removal. The limited but promising results using pure O_2_ also indicate the potential for more targeted dosing in systems with high H_2_S loads. The chemical complexity of manure-based substrates introduces different challenges, especially due to their variable sulfur content and microbial communities, which may respond differently to microaeration.

Manure-based substrates, such as dairy, poultry, and swine manures, also show strong H_2_S mitigation under microaerobic conditions, though fewer studies are available compared to wastewater systems. As seen in Table 2, all the manure-based studies used ambient air for their microaeration treatments. In batch systems, one study applying 7 to 50 mL air/g VSreported H_2_S removal ranging from 28% to 58% [72], while another study using a rate of 66.7 mL air/L/min achieved up to 70.2% removal [96]. No semi-continuous trials were identified for microaeration of manure. In a continuous system, daily aeration of 20 m^3^ air/day resulted in a 68.2% reduction in H_2_S concentration [97]. All studies were conducted under mesophilic conditions. While removal efficiencies in these studies are somewhat lower than those reported for sludge-based systems, they still demonstrate meaningful reductions in H_2_S under relatively low air input. These findings suggest that even modest oxygen additions can stimulate sulfur-oxidizing activity in manure-rich digesters, though the effectiveness may depend on specific digester conditions, including sulfur content and microbial community composition.

Although this review primarily focuses on agricultural byproducts, microaeration has also been applied in digesters treating food waste and lignocellulosic substrates. It was reported that microaeration led to a significantly more diverse bacterial community as compared to anaerobic conditions. A higher relative abundance of Firmicutes enhanced the metabolic capacity of the acidogenic reactor, enabling it to utilize a wider range of substrates and resulting in greater COD solubilization and VFA production under microaerobic conditions [98].

Across the studies reviewed, microaeration has proven to be an effective strategy for reducing H_2_S concentrations during anaerobic digestion, particularly in wastewater and sludge-based systems. Continuous reactors were the most frequently studied and consistently showed high H_2_S removal efficiencies, often exceeding 90% even under relatively low ambient air input [46,88,89,92,93]. In contrast, batch and semi-continuous systems were less commonly evaluated, and while they demonstrated substantial reductions in H_2_S, the range of outcomes was broader and more variable [72,86,87]. Most studies were conducted under mesophilic conditions, leaving thermophilic performance underexplored, with only one study reporting 75 to 100% removal under thermophilic conditions [94]. While ambient air was the predominant aeration source, limited trials using pure oxygen also showed promising results, with greater than 99% H_2_S removal reported in both semi-continuous and continuous systems [91,95]. Manure-based digesters responded positively to microaeration but generally exhibited lower removal rates than sludge-based systems, likely due to greater substrate heterogeneity and differences in microbial dynamics [72,96,97]. Together, these findings support the adaptability of microaeration across diverse system configurations and highlight the need for further research on underrepresented reactor types and temperature regimes.

**Table 2:** Effects of microaeration on hydrogen sulfide production during anaerobic digestion under various conditions.

Digester Type	Materials	Digestion Temperature	Working Volume	Aeration Rate	Effect on H_2_S	Reference
Batch	Sludge	37 °C	2.7 L	1 L air/day	73–99% removal	[86]
Batch	Sludge	30 ± 1 °C	1 L	0.2 mg air/L	35–80% removal	[87]
Batch	Chicken manure	37 ± 1 °C	120 mL	7–50 mL air/gVS	28–58% removal	[72]
Batch	Swine wastewater	35 ± 1 °C	4 L	66.7 mL air/L/min	<70.2% removal	[96]
Semi-continuous	Sludge	35 ± 0.2 °C	50 L	0.14 mL O_2_/s	58–99% reduction	[36]
Continuous	Sludge	35 ± 1 °C	200 L	0.2–0.25 Nm^3^ O_2_/m^3^	>99% removal	[95]
Continuous	Dairy manure	35 °C	338 m^3^	20 m^3^ air/day	68.2% reduction	[97]
Continuous	Sludge	35 °C	50 L	4.4–6.2 NL air/m^3^/day	90% removal	[92]
Continuous	Sludge	---	---	0.28–6.00 m^3^ air/h	73.8–99.1% removal	[46]
Continuous	Sludge	40 °C	120 L	2–400 mL air/min	87–97.5% removal	[90]
Continuous	Sludge	55 °C	<500m^3^	0.09–0.77 kNm^3^ air/day	75–100% removal	[94]
Continuous	Wastewater	20–22 °C	3.8 L	10–20 mL air/min	60.6–100% removal	[91]
Continuous	Sludge	33–37.5 °C	2450–3300 m^3^	2.5–14 m^3^ air/h	50–87% removal	[88]
Continuous	Wastewater	15–25 °C	24 m^3^	8.2–32.7 m^3^ air/day	>99% removal	[93]
Continuous	Sludge	35 °C	6965 m^3^	2.5–5 m^3^ air/h for 49 days	13–20% removal	[89]

### 6.3. Process Stability and Digestion Kinetics

Other effects of microaeration are summarized in Table 3. Food waste digesters have shown variable but often positive shifts in process efficiency when treated with microaeration. Due to its high biodegradability and rapid acidification potential, food waste benefits from enhanced hydrolysis and stabilization under controlled oxygen exposure. In batch systems, pretreatment with 5 L air/h for 24 h led to a 14.5% to 37.6% reduction in volatile fatty acids (VFAs) and a 45.8% increase in net energy generation [85]. Another batch study using injections of 5 to 20 mL O_2_ as a pretreatment reported changes in chemical oxygen demand (COD) removal ranging from a 28% decrease to a 23% increase, highlighting sensitivity to dosing levels [99]. In a semi-continuous system, a 400 L air pretreatment improved COD removal by 25% and resulted in a 10% increase in VFA concentrations, indicating enhanced solubilization with potential tradeoffs in acidogenesis [100]. All studies were conducted under mesophilic conditions. No studies evaluated continuous systems using aeration pretreatment for food waste. These findings suggest that pretreatment with oxygen can enhance solubilization and hydrolysis, but overexposure may shift fermentation dynamics or inhibit methanogenesis if not optimized.

In contrast, when microaeration is applied during digestion, food waste systems tend to show stronger improvements in decomposition kinetics. In batch systems, one thermophilic study applying 2.5 to 20 mL air/day observed volatile solid removal increases ranging from 1.6% to 10.1%, indicating enhanced substrate degradation [20]. A separate mesophilic batch experiment using a specific aeration rate of 274 L air/kg TS/day reported a 36% increase in VFA production, suggesting intensified acidogenesis during the digestion phase [101]. No semi-continuous or continuous studies were identified for food waste digestion under microaerobic conditions during the active digestion phase. These results highlight how oxygen delivered during the active digestion phase can stimulate facultative and hydrolytic microbes, accelerating substrate breakdown. However, the magnitude of these benefits appears highly sensitive to dosing intensity, timing, and reactor design.

Microaeration has also been explored in wastewater and sludge-based systems as a strategy to improve digester performance and hydrolysis efficiency. In batch systems, substantial gains have been reported. One study applying 254 L air/kg TS/day observed a 200% increase in decomposition and a 250% increase in VFA concentration [102], while another using 4.6 to 15.4 mL air/L/min achieved between 46% and 123% increases in VFAs [103]. In a semi-continuous reactor, an oxygen dose ranging from 5 to 150 mg O_2_/L resulted in an improvement in hydrolysis efficiency ranging from 39.9% to 48.7% [81]. Continuous flow systems have shown similarly promising outcomes, with one study reporting an 86.9% increase in COD at an aeration rate of 0.5 m^3^ air/h over 72 h [104], and another demonstrating a 59.2% improvement in sludge reaction using 3 to 4 mg O_2_/L [94]. All studies were conducted under mesophilic conditions. These results demonstrate the capacity of controlled oxygen exposure to enhance breakdown rates and solubilization in sludge systems, though outcomes remain sensitive to dosing strategy.

Across food waste and wastewater systems, microaeration has demonstrated clear potential to improve overall digester performance, particularly by enhancing hydrolysis, solubilization, and intermediate metabolite production. Batch systems dominate the existing literature, especially in food waste applications, with fewer examples of semi-continuous and continuous designs, most of which are concentrated in wastewater treatment contexts [81,85,100,102,104]. These differences in reactor type likely influence oxygen transfer dynamics and microbial response, affecting the magnitude and consistency of observed performance gains. Nearly all studies were conducted under mesophilic conditions, with only one thermophilic trial identified, which showed moderate improvements in VS removal following controlled oxygen addition to a food waste digester [20]. In wastewater systems, continuous and semi-continuous reactors have shown substantial increases in COD removal, sludge reduction, and VFA accumulation when aeration rates were properly optimized [81,102,103,104,105]. These findings underscore the importance of tailoring microaeration strategies to the specific reactor configuration and temperature regime, as system design, temperature and oxygen dosing collectively determine the balance between enhanced substrate degradation and potential microbial inhibition.

Although microaeration enhances substrate solubilization and hydrolysis, excessive oxygen exposure can temporarily favor acidogenesis over methanogenesis. Part of the organic carbon may be oxidized to CO_2_ or accumulate as VFAs, reducing the fraction converted to CH_4_ [8,42]. Maintaining oxygen input within a narrow microaerobic range supports both solubilization and stable methanogenic activity, emphasizing the importance of controlled dosing in achieving balanced process performance [9,71].

## 7. Process Modeling and Predictive Tools

Mathematical modeling has long played a central role in understanding and optimizing the AD process. One of the most widely used frameworks is the Anaerobic Digestion Model No. 1 (ADM1), developed by the International Water Association (IWA) in 2002 [106]. ADM1 simulates the biochemical and physicochemical processes involved in AD, including hydrolysis, acidogenesis, acetogenesis, and methanogenesis, along with pH, gas transfer, and ion balance. It has become the standard platform for predicting system performance, aiding in design, control, and scale-up of full-scale digesters [106,107].

However, conventional ADM1 does not account for oxygen transfer or oxidation pathways, limiting its use in systems that incorporate microaeration. In response, recent studies have modified ADM1 to simulate the impacts of low-level aeration, enabling predictions of key outcomes such as CH4 yield, H2S removal, and process stability under microaerobic conditions [108,109,110,111]. These adaptations often introduce new microbial populations, oxygen kinetics, and redox-sensitive reactions to capture dual anaerobic–microaerobic interactions that occur in aerated systems [108,109]. For instance, ADM1 Sulfur Oxidation (ADM1-SO) models have incorporated pathways for H_2_S oxidation by facultative and autotrophic SOB [112,113,114]. These extensions simulate how small aeration inputs convert H_2_S into S or SO_4_, significantly improving predictive accuracy for biogas desulfurization strategies. Studies using this model framework have demonstrated its effectiveness in predicting both H_2_S removal and the impact of dosing rate on microbial community shifts and gas quality [113,114].

Other modeling tools have taken a broader optimization-based approach. Dynamic situation models coupled with control algorithms have been developed to predict threshold oxygen concentrations that enhance digester performance without inhibiting CH_4_ production [83,111,115,116]. These tools can simulate various dosing strategies, including continuous, intermittent, and step-wise additions, to identify optimal aeration schedules for specific substrates and reactor types. This approach has proven especially valuable for systems digesting food waste or high-sulfur substrates, where dosing precision is critical [78,83,112].

In up-flow anaerobic sludge blanket (UASB) systems, calibrated ADM1 extensions have been used to model spatial heterogeneity in oxygen diffusion and microbial distribution [108,109,117]. These spatially resolved models are particularly useful for stimulating partial oxygen penetration and the resulting stratified microbial zones within the reactor. They provide insights into how microaeration impacts reactor hydrodynamics and sludge granule integrity, helping to inform reactor design and operational strategies [108,109,118,119].

Reliable simulation of microaeration depends on careful model calibration and validation against experimental data. Most ADM1-based models are calibrated using laboratory or pilot-scale measurements of biogas composition, H_2_S removal efficiency, VFA concentration, and redox potential under controlled aeration [111]. Validation commonly involves comparison with independent datasets from pilot or full-scale digesters to assess predictive accuracy across operational conditions [113]. However, reliability decreases under dynamic events such as fluctuating aeration rates or organic shock loads, where oxygen transfer kinetics and microbial adaptation are difficult to parameterize [114]. Recent studies incorporating real-time feedback control or data-driven surrogates have improved model responsiveness, but further calibration under transient conditions remains essential for robust performance prediction. Some researchers have explored machine learning and data-driven approaches to complement or extend mechanistic models [120,121]. These methods use empirical data to train predictive algorithms that can estimate performance metrics such as VFA accumulation, CH_4_ yield, or H_2_S levels based on operational inputs like oxygen dosing rate, substrate type, and HRT. While still emerging, these tools offer promising avenues for real-time monitoring and adaptive control in full-scale operations [121,122].

Overall, the growing suite of process modeling and predictive tools highlights the importance of simulation-based design in advancing aeration strategies. While substrate-specific calibration remains a challenge, ongoing refinement of these tools enhances their generalizability and utility in diverse digester configurations [122].

## 8. Cost–Benefit Analysis of Microaeration Systems

Microaeration introduces additional capital and operational costs into AD systems, primarily due to the need for aeration equipment, control systems, and potential retrofitting of existing reactors. However, these costs are often offset by improvements in process performance and reductions in downstream treatment expenses [8,21,61]. 

For instance, studies have reported that moderate aeration can significantly reduce H_2_S levels, which decreases corrosion in gas handling infrastructure and lowers the need for costly chemical scrubbers or iron salts [5,91,93]. Costs for chemical scrubbing alone can range from $1.23 to $2.38/m^3^ biogas, while microaeration treatment costs are significantly lower, ranging from just $0.0015 to $0.0045/m^3^ biogas [5]. The economic benefits from extended equipment lifespan and reduced maintenance frequency can be substantial, especially in systems with high sulfur loads [5,123].

In small farm-scale digesters, the marginal cost of installing air pumps and control valves represents less than 3–5% of total capital expenditure [5], while in large municipal reactors, operational energy requirements are often below 0.1% of total process energy demand [124]. Despite these advantages, several hidden or indirect costs should be considered. Routine maintenance of oxidation-reduction potential and dissolved-oxygen sensors can increase operational labor, and biofilm accumulation on sensors or diffusers may require periodic cleaning or replacement [48]. Intermittent aeration can occasionally trigger foaming, requiring antifoam dosing or headspace management [125]. Furthermore, excessive oxygen exposure may stimulate nitrous oxide (N_2_O) emissions, a factor that is rarely quantified but could influence the overall environmental balance [42]. Where available, life-cycle assessments (LCAs) support the economic findings, showing that microaeration achieves lower global-warming potential and acidification impacts than chemical or biological scrubbing when electricity use remains minimal [36,123,125]. These results suggest that while microaeration delivers clear cost advantages, integrating full-system evaluations that include sensor maintenance, auxiliary energy inputs, and potential gaseous by-products is essential for a realistic cost–benefit assessment [123]. From a biogas quality perspective, microaeration may also improve CH_4_ content by reducing impurities such as H_2_S, thereby increasing the value and marketability of the gas [36]. In some cases, higher CH_4_ yields associated with enhanced hydrolysis or toxicity mitigation further improve energy recovery, providing a secondary revenue stream [43,72,124]. While energy input for delivery must be considered, several studies have shown that optimized dosing results in negligible increases in energy demand relative to the process gains achieved [123,124].

Comprehensive cost–benefit analyses have begun to model these trade-offs under various scenarios of substrate type, reactor configuration, and dosing strategies. These analyses often conclude that microaeration becomes economically favorable when targeting sulfide-prone substrates, integrated into existing infrastructure or when paired with energy recovery systems [5,123,126,127]. Nonetheless, site-specific evaluation remains crucial, as over-aeration or poor control can negate benefits and increase operational risks [5,8,126]. As modeling tools and monitoring technologies improve, real-time optimization of dosing may further increase the financial viability of microaeration in both small and large-scale applications [120,121,127].

## 9. Future Perspectives

While microaeration has demonstrated clear potential to enhance anaerobic digestion by improving CH_4_ yield, reducing H_2_S, and boosting overall process efficiency, its broader application remains limited by several knowledge gaps. Further research is needed to better understand and optimize microaeration across diverse substrates, reactor designs, and operational scales. The following areas represent key priorities for future investigation:

- Microbial mechanisms and community shifts
  More research is needed to clarify how low oxygen levels influence microbial community structure, particularly the activity of SOB and facultative organisms during long-term operation.
- Oxygen transfer and dosing optimization
  Studies should focus on quantifying oxygen mass transfer rates in different reactor types and under varying conditions to improve dosing strategies and prevent over-aeration.
- Impacts on digestate quality and emissions
  The long-term effects of microaeration on digestate composition, nutrient availability, and emissions of nitrous oxide (N_2_O) require further study to ensure environmental compliance.
- Emerging contaminants
  Recent findings indicate that anaerobic digestion can transform certain emerging contaminants, such as tetrabromobisphenol A, through partial reductive debromination to bisphenol A [128], which highlights the need for more research regarding the removal of emerging contaminants.
- Integration with automation and monitoring systems
  Research is needed on how to incorporate real-time monitoring and control systems for precise oxygen delivery, especially in large-scale or continuously fed digesters.
- Economic and life cycle assessments
  Comprehensive cost–benefit analyses and life cycle assessments across different substrates and regions are essential to determine the economic viability and environmental sustainability of microaeration.
- Application across understudied substrates
  More data is needed for substrates such as industrial organic waste, agricultural residues beyond manure, and high-fat or protein-rich waste streams to understand the broader applicability of microaeration.

## 10. Conclusions

Microaeration has emerged as a practical and low-cost strategy to address hydrogen sulfide accumulation and improve process stability in anaerobic digestion systems treating manures, lignocellulosic residues, food waste, and sewage sludge. Studies conducted between 2015 and 2025 consistently demonstrate significant H_2_S removal efficiencies, often exceeding 90% in continuous reactors supplied with low airflow rates. Additional benefits include enhanced hydrolysis of recalcitrant substrates, mitigation of sulfide toxicity, and moderate improvements in methane yield, typically ranging from 5 to 20%. However, responses vary widely depending on substrate composition, reactor type, operating temperature, and aeration control. Excess oxygen dosing remains a critical risk, as it can suppress methanogenesis, increase CO_2_ production, and introduce safety concerns when oxygen accumulates in the gas phase.

Process monitoring based on oxidation-reduction potential provides a useful proxy for maintaining favorable redox conditions, while dissolved oxygen measurements remain limited by fouling and low resolution at trace levels. Advances in modeling, particularly modifications to ADM1 that incorporate oxygen transfer and sulfur oxidation pathways, have improved predictive accuracy for both methane yield and biogas desulfurization under microaerobic conditions. Economic assessments indicate that microaeration delivers substantial cost advantages compared to conventional chemical scrubbing, with treatment costs several orders of magnitude lower.

Overall, microaeration represents a versatile and scalable tool for improving biogas quality. Future development should prioritize automation through real-time control of oxidation–reduction potential and dissolved oxygen, enabling adaptive aeration strategies that respond to dynamic loading conditions. Integration with other biogas upgrading technologies, such as membrane separation, biochar filtration, or biological methanation, could further enhance gas purity and energy recovery at full scale. Coupling automated microaeration with digital monitoring and life-cycle modeling will be essential for translating laboratory advances into reliable, large-scale operation across agricultural systems.

## Figures and Tables

**Table 3:** Effects of microaeration on digester performance during anaerobic digestion under various conditions.

Reactor Type	Materials	Digestion Temperature	Working Volume	Aeration Rate	Effect	Reference
Batch	Corn straw	55 °C	200 mL	2.5–20 mL air/day	1.6–10.1% increase VS removal	[20]
Batch	Synthetic food waste	30 ± 2 °C	500 mL	274 L air/kgTS/day	36% increase VFA	[101]
Batch	Synthetic food waste	35 ± 1 °C	1 L	5 L air/h for 24 h	14.5–37.6% decrease VFA 45.8% increase energy generation	[85]
Batch	Municipal solid waste	38 ± 1 °C	1.5 L	254 L air/kgTS/day	200% increase decomposition 250% increase VFA	[102]
Batch	Food waste	37 °C	1 L	5–20 mL O_2_ pretreatment	28% decrease-23% increase COD removal	[99]
Batch	Sludge	25 °C	1 L	4.6–15.4 mL air/L/min	46–123% increase VFA	[103]
Semi-continuous	Food waste	30 ± 2 °C	20 L	400 L pretreatment	10% increase VFA 25% increase COD removal	[100]
Semi-continuous	Blackwater	22 °C	1.5 L	5–150 mg O_2_/L	39.9–48.7% increase hydrolysis	[81]
Continuous	Wastewater	---	2 L	0.5 m^3^ air/h for 72 h	86.9% increase COD	[104]
Continuous	Wastewater	---	50 L	3–4 mg O_2_/L	59.2% increase sludge reduction	[105]

## Data Availability

Since this is a review manuscript no new data were created in this study. Data sharing is not applicable to this article.

## Abbreviations

The following abbreviations are used in this manuscript:

AD	Anaerobic digestion
ADM1	Anaerobic digestion model no. 1
ADM1-SO	Anaerobic digestion model no 1: sulfur oxidation
COD	Chemical oxygen demand
DO	Dissolved oxygen
HRT	Hydraulic retention time
IWA	International water association
ORP	Oxidation-reduction potential
ppm	Parts per million
SOB	Sulfur oxidizing bacteria
TS	Total solids
UASB	Upflow anaerobic sludge blanket
VFA	Volatile fatty acid
VS	Volatile solids
